# Clinical effect of endovascular repair of complex aortic lesions using optimized Octopus surgery

**DOI:** 10.3389/fbioe.2023.1240651

**Published:** 2023-07-20

**Authors:** Yanfeng Sun, Yang Zhang, Xiwei Sun, Hang Yin, Shuai Wang, Xiao Li, Zhongying Wang, Sean X. Luo, Zhihua Cheng

**Affiliations:** Department of Vascular Surgery, General Surgery Center, The First Hospital of Jilin University, Changchun, China

**Keywords:** Octopus technique, pararenal aortic aneurysm, thoracoabdominal aortic aneurysm, aortic dissection, gutter endoleak, visceral artery branches

## Abstract

**Objective:** Complex aortic lesions, especially those involving branches of the visceral artery, remain a challenge to treat. A single-center study using the Octopus technique to evaluate the safety and short-term effects of endovascular repair of complex aortic lesions was reported and documented.

**Methods:** The data of six cases who underwent optimized Octopus surgery in our center from August 2020 to February 2022 were analyzed retrospectively. The choice of operation scheme, operation time, operation complications, and follow-up data were analyzed among them.

**Results:** The average age of the six patients undergoing optimized Octopus surgery was 55.1 ± 17.2 years. Two cases were diagnosed as pararenal aortic aneurysms; four cases were aortic dissection involving the visceral artery. All cases achieved technical success; all visceral arteries were reconstructed as planned. A total of 17 visceral arteries were planned to be reconstructed; five celiac arteries were embolized. Three cases of gutter endoleak were found during the operation without embolization but with follow-up observation. There were two cases of slight damage to renal function and two cases of perioperative death. Other complications, such as intestinal ischemia and spinal cord ischemia, did not occur. Follow-up ranged from 6 months to 30 months. One patient died of gastrointestinal bleeding 6 months after the operation. At the 6 months follow-up, computed tomographic angiography showed that all internal leaks had disappeared. The patency rate of the visceral artery was 100%, and no complications, such as stent displacement and occlusion, occurred during the follow-up period.

**Conclusion:** With fenestrated and branched stent grafts technology not widely available, and off label use not a viable option, Octopus technology for treating complex aortic lesions should be considered. The Octopus technique is an up-and-coming surgical method, but we should recognize its operation difficulty, operation-related complications, and long-term prognosis. We should pay attention to and continue to optimize Octopus technology.

## Introduction

The expression “complex aortic lesions” (CALs) mainly refers to aortic aneurysm and aortic dissection involving the visceral artery. At present, the gold standard for the treatment of these diseases is open surgery. Although open surgery has been improved in perioperative nursing and surgical instruments in recent years, there are still high perioperative mortality and complications in the open surgical treatment of CALs. A meta-analysis of 7833 cases of open surgical repair of thoracoabdominal aortic aneurysm (TAAA) from 2000 to 2010 found that the incidence of pulmonary insufficiency was 31%, the incidence of renal failure was 19.8%, the average hospital mortality was 10%, and the rate of spinal cord ischemia was 7.5% (Piazza and Ricotta, 2012). Therefore, treating CALs remains a challenge.

More and more surgeons and patients have accepted endovascular repair of aortic lesions because of its advantages, such as less trauma and quick recovery. However, the endovascular repair of CALs has always been a complex problem. With the development of technology and innovative ideas, Chuter et al. introduced the first visceral artery stent implantation system in 2001 (Chuter et al., 2001), which was used to reconstruct the visceral branches of TAAA to retain the function of essential organs. In recent years, many innovative endovascular treatments have been proposed, for example, sandwich technology (Lobato and Camacho-Lobato, 2013), chimney technology (Richardson et al., 2011; Kolvenbach, 2013), periscope technology (O’Neill et al., 2006; Rancic et al., 2010), and fenestrated and branched stent grafts technology (Sweet et al., 2009; Verhoeven et al., 2009; Gallitto et al., 2022), which have achieved good results, but there are still many cases that cannot satisfy the requirements of endovascular repair of CALs.

Octopus technology uses existing covered stents for sophisticated assembly designs to treat CALs without modifying stents. In 2011, Kasirajan (2011) first reported using Octopus technology for the endovascular treatment of CALs. In 2012, Silveira et al. (2012) described the classic Octopus surgery. The following introduces our center’s experience in the application of Octopus technology in the treatment of CALs.

## Materials and methods

This study uses descriptive statistical methods for statistical analysis. This study is in line with the Helsinki Declaration and has been approved by the local institutional review committee. All patients and their families agreed to accept our Octopus technical plan and signed the relevant, informed consent form before the operation.

### Patient characteristics

Six patients (three males) who received Octopus treatment from August 2020 to February 2022 were included. All of these cases were examined using computed tomographic angiography (CTA) before the operation, two cases were diagnosed as pararenal aortic aneurysms, and four cases were diagnosed as aortic dissection involving visceral artery branches. Basic information about patients, primary diseases, perioperative data (including operation time, blood loss, blood transfusion, complications, etc.), and follow-up data were recorded (see Tables 1, 3, 4).

**TABLE 1:** Clinical data of patients with complex aortic lesions.

Case	Sex	Age (years)	Diagnosis	Basic diseases	Surgical history
1	M	57	Aortic dissection	Hypertension, Cerebral haemorrhage	History of ERAD, surgical history of a cerebral haemorrhage
2	F	75	PRAA	Hypertension, Renal insufficiency	No
3	F	53	Aortic dissection	Hypertension	History of ERAD
4	M	53	Aortic dissection	No	No
5	F	68	PRAA	No	No
6	M	25	Aortic dissection	Hypertension, Uremia, CLBBB, Hyperthyroidism, Hyperkalemia	No

M, male; F, female; PRAA, pararenal aortic aneurysm; CLBBB, complete left bundle branch block; ERAD, endovascular repair of aortic dissection.

### Operation process

After applying general anesthesia, bilateral common femoral arteries and bilateral brachial artery pathways were established, and systemic heparinization was performed before endovascular treatment. All operations were performed in the digital subtraction angiography operating room. An Excluder bifurcation stent (W.L. Gore & Associates, Newark, DE, United States) with appropriate diameter was placed in the descending aorta through one common femoral artery pathway (because the maximum diameter of the existing Excluder bifurcation stent is 35 mm, if it cannot reach 1.2 times the diameter of the descending aorta release position, a CUFF stent is placed at the proximal end of the bifurcation stent to complete proximal closure and reduce the incidence of stent displacement and type Ia endoleak). The lower edge of the short leg of the bifurcation stent was located above the celiac artery (CA), and the lower edge of the long leg of the bifurcation stent was placed above the left renal artery (LRA). A balloon dilated the proximal anchoring area of the bifurcation stent. Then, the guide wire combined with the catheter was passed through the left brachial artery and bifurcation stent short leg into the CA for coil embolization to reduce the occurrence of type II endoleak.

Viabahn stents (W.L. Gore & Associates) of appropriate diameter were implanted. Through the contralateral common femoral artery pathway, the guide wire combined with the catheter entered into the short leg of the bifurcation stent, and an iliac leg-covered stent (W.L. Gore & Associates) was implanted. Through the bilateral brachial artery pathway, the guide wires combined with the catheters entered the right renal artery (RRA) and superior mesenteric artery (SMA) through a bifurcation stent short leg and iliac leg-covered stent, respectively. The Viabahn stents in the RRA and SMA were released simultaneously and extended into the iliac leg-covered stent with at least a 3 cm overlap. After the reconstruction of the two visceral arteries, balloon dilatation was performed. After dilatation, the best shape of the two Viabahn stents in the iliac leg-covered stent was the shape of, which reduced the occurrence of gutter endoleak to the greatest extent.

**FIGURE 1:**
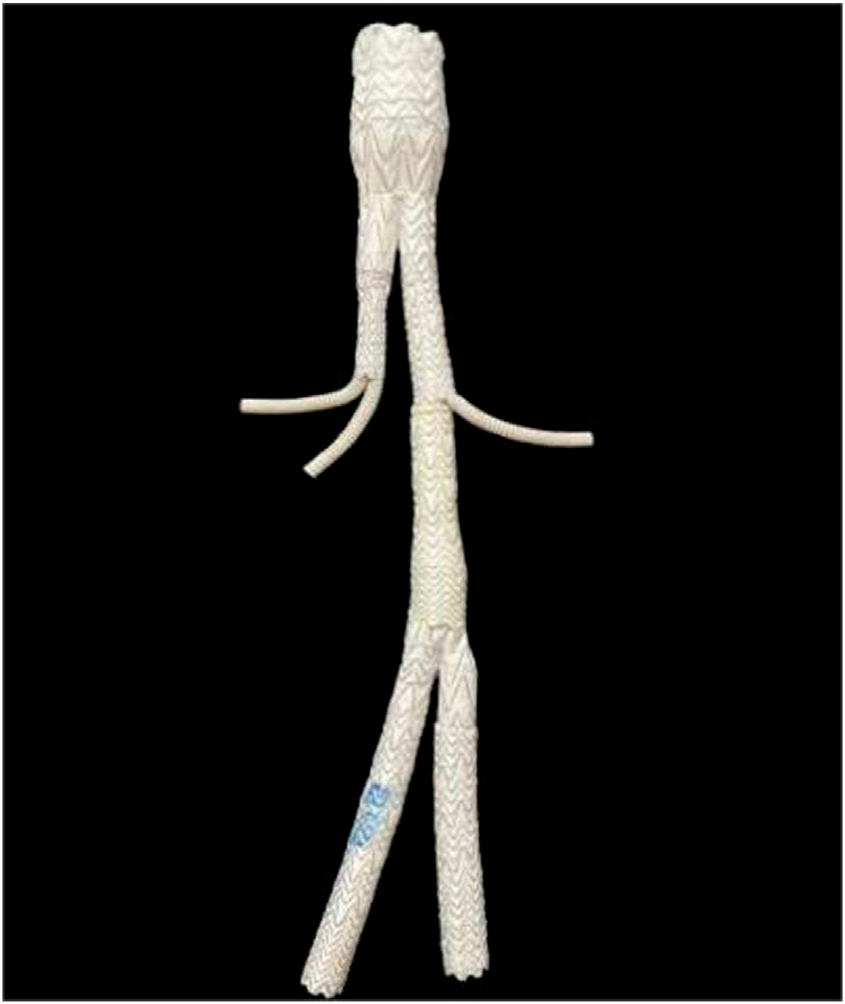
Optimize octopus technology.

Through the left brachial artery, the guide wire combined with the catheter entered the LRA through the long leg of the bifurcation stent, implanted a Viabahn stent of appropriate diameter and extended into at least 3 cm in the long leg, and implanted a trumpet leg covered stent of appropriate diameter into the extended leg through the pathway in which the bifurcation stent was previously placed, overlapping 3 cm with the extended leg and releasing it. Another Gore Excluder bifurcation stent was inserted into the trumpet leg, and the remaining operation was carried out according to the standard endovascular aneurysm repair; that is, the short leg and the long leg are connected with the iliac leg covered stent and extended into the bilateral common iliac artery (according to the specific conditions of the patient, if the iliac artery is dilated, the stent can extend into the external iliac artery and embolize the internal iliac artery). Balloon dilatation stents overlap, and stent and vascular areas overlap. High-pressure syringe angiography checks whether stent displacement, thrombosis formation in the stent, or internal leakage occurred. If the operation goes well, the four vascular pathways are closed, and the operation is over (Figure 1).

### Postoperative nursing and follow-up

After the operation, according to the patient’s specific conditions, they can be transferred to the intensive care unit (ICU) for further support of vital signs. On the first day after an operation, low molecular weight heparin sodium was administered for sufficient anticoagulation according to the patient’s body weight to prevent in-stent thrombosis. After discharge, oral aspirin and clopidogrel were supplied to maintain the patency of the stent. CTA or ultrasound re-examination was performed at the first week, 6th month, 12th month, and every year thereafter.

## Results

**FIGURE 2:**
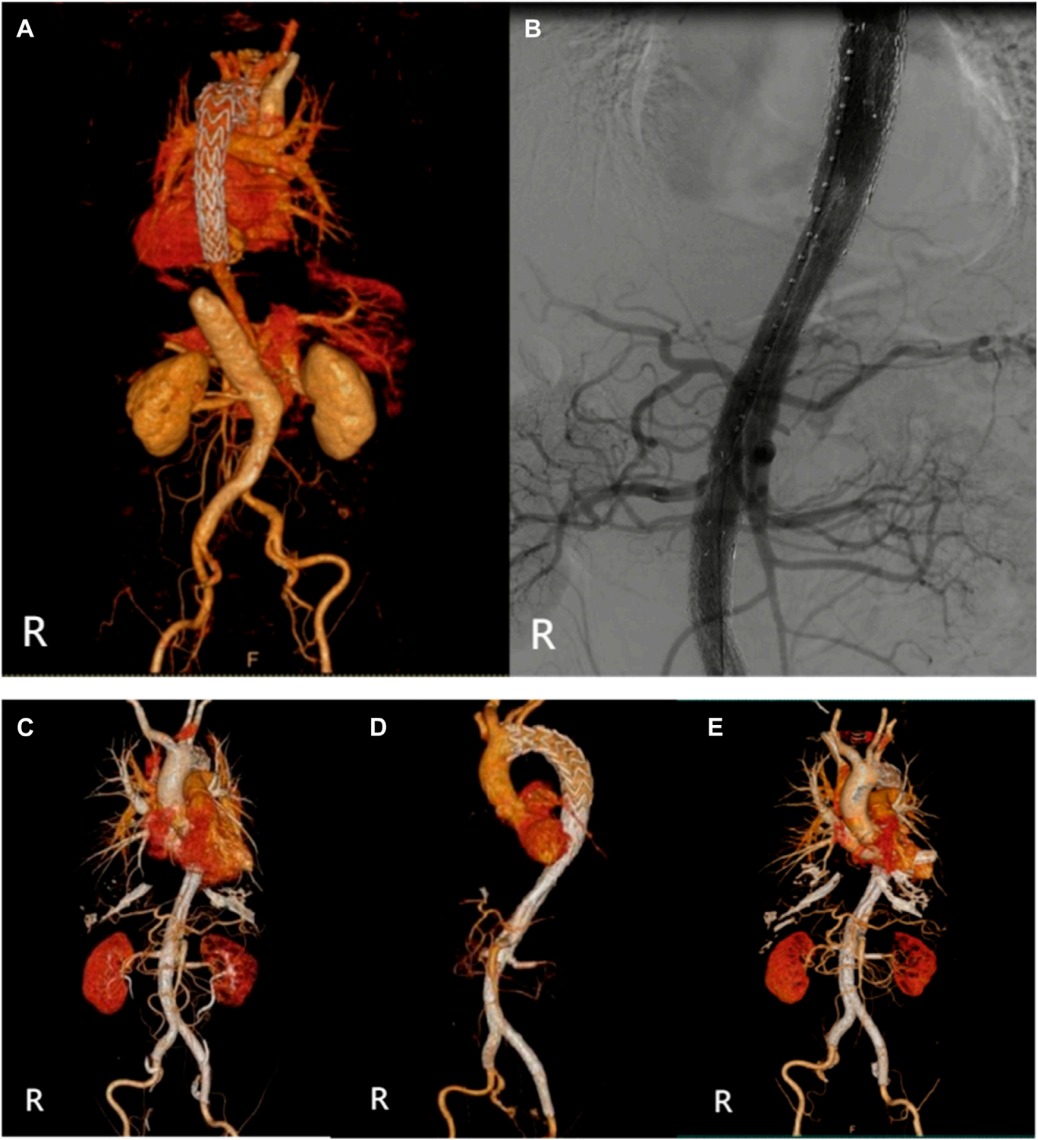
(Case 1) **(A)** Preoperative CTA. **(B)** Intraoperative imaging. **(C)** Postoperative CTA (1st week). **(D)** Postoperative CTA (6th month). **(E)** Postoperative CTA (24th month).

**FIGURE 3:**
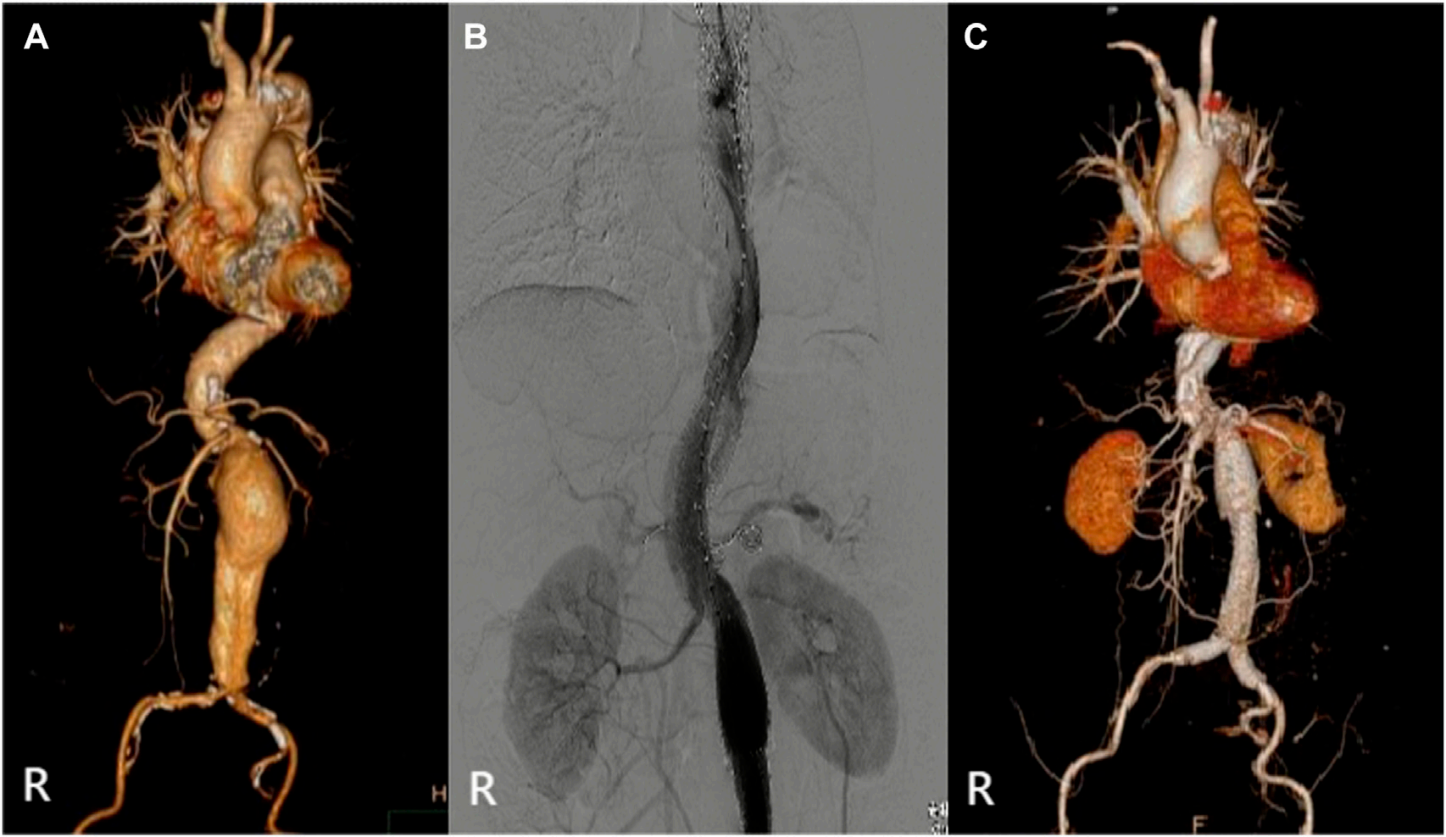
(Case 2) **(A)** Preoperative CTA. **(B)** Intraoperative imaging. **(C)** CTA (6th month).

**FIGURE 4:**
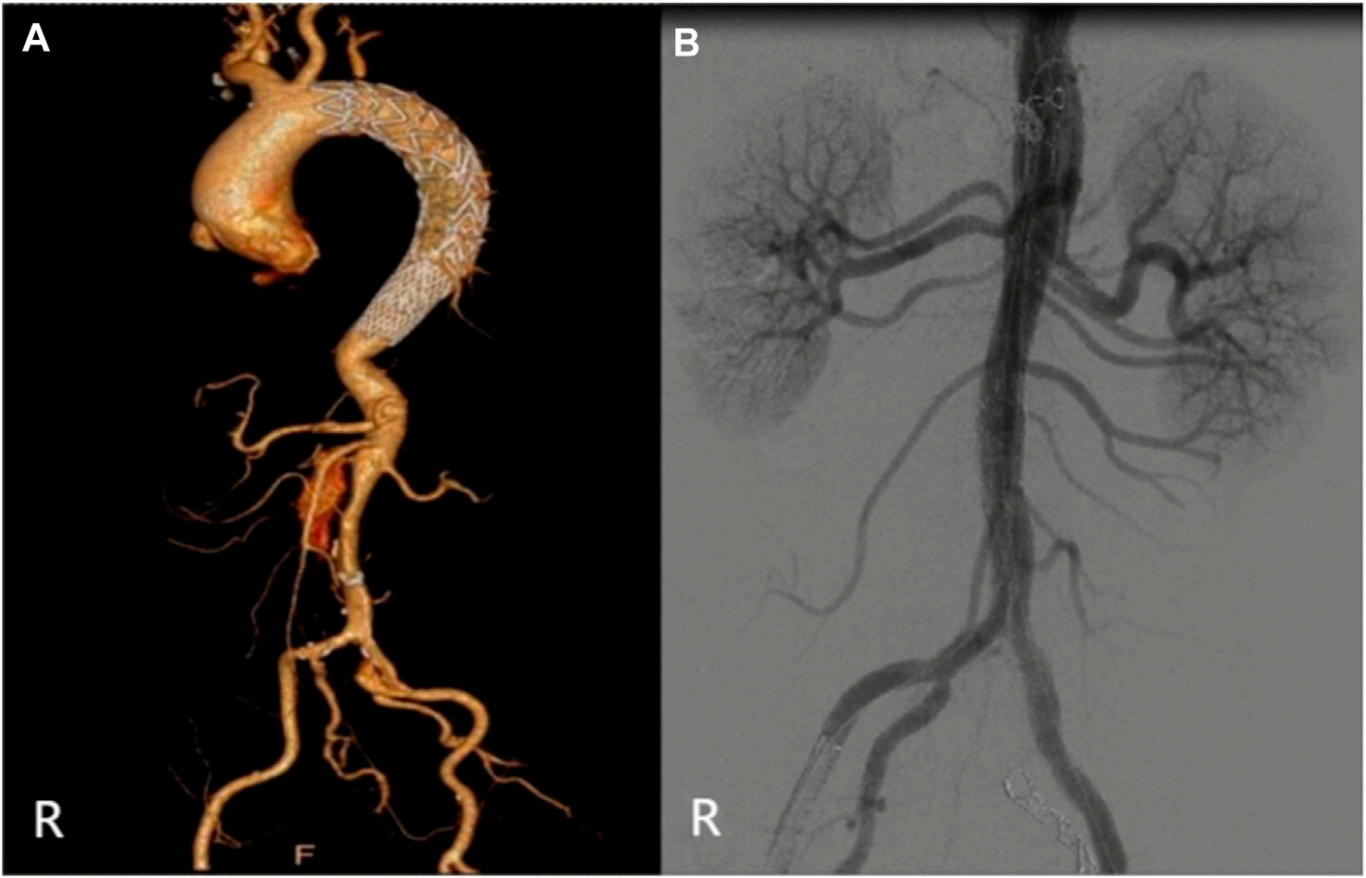
(Case 3) **(A)** Preoperative CTA. **(B)** Intraoperative imaging.

**FIGURE 5:**
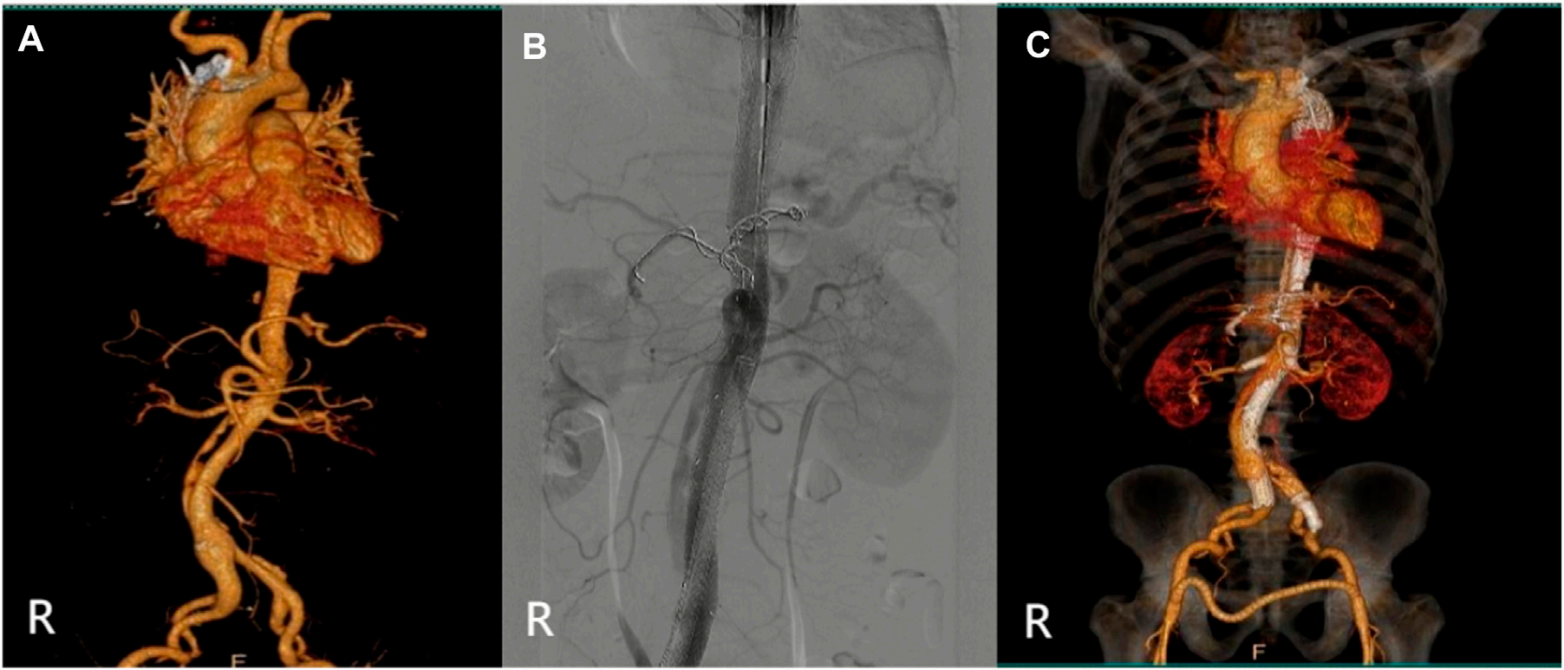
(Case 4) **(A)** Preoperative CTA. **(B)** Intraoperative imaging. **(C)** CTA (1st week).

**FIGURE 6:**
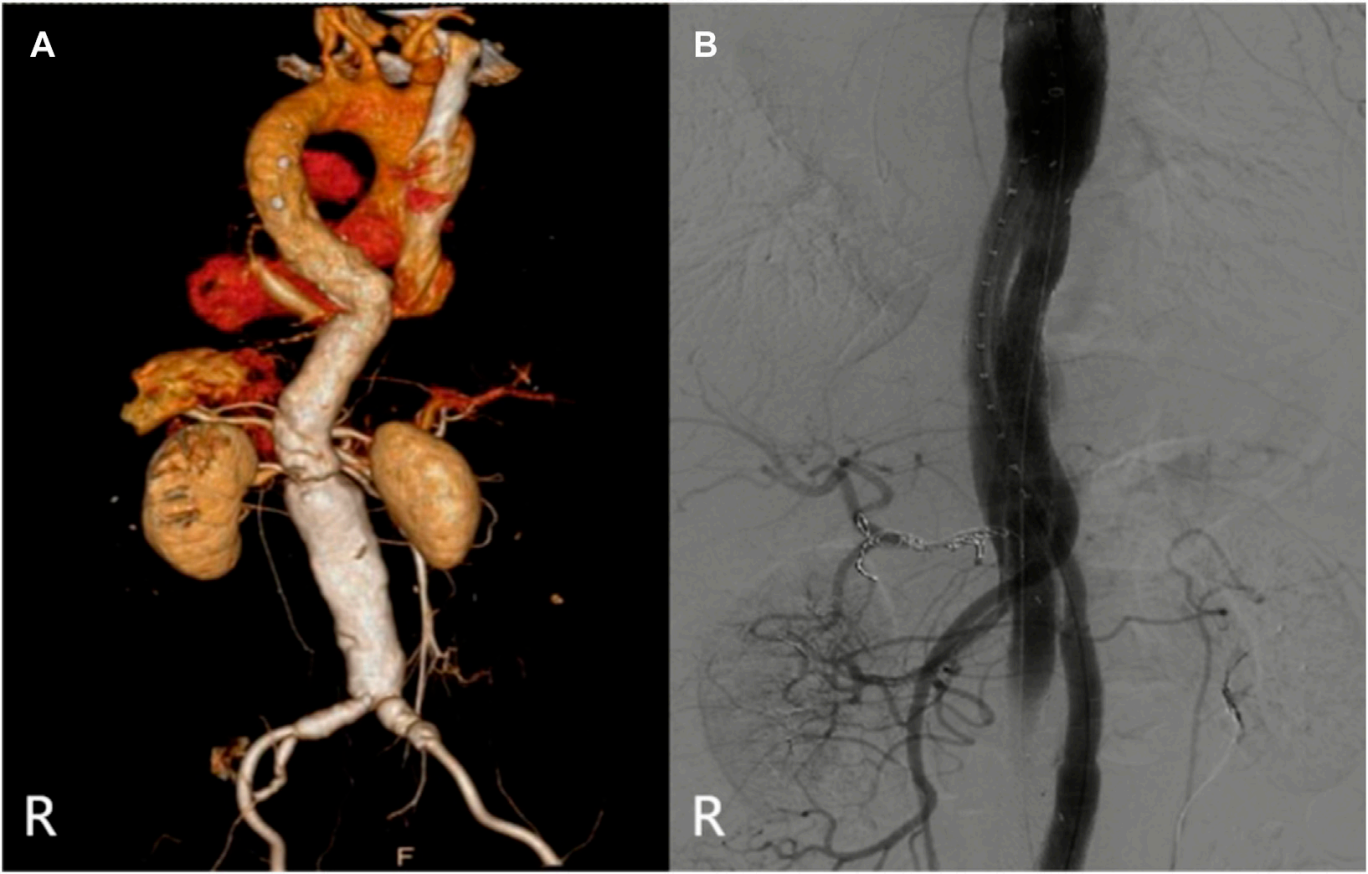
(Case 5) **(A)** Preoperative CTA. **(B)** Intraoperative imaging.

**FIGURE 7:**
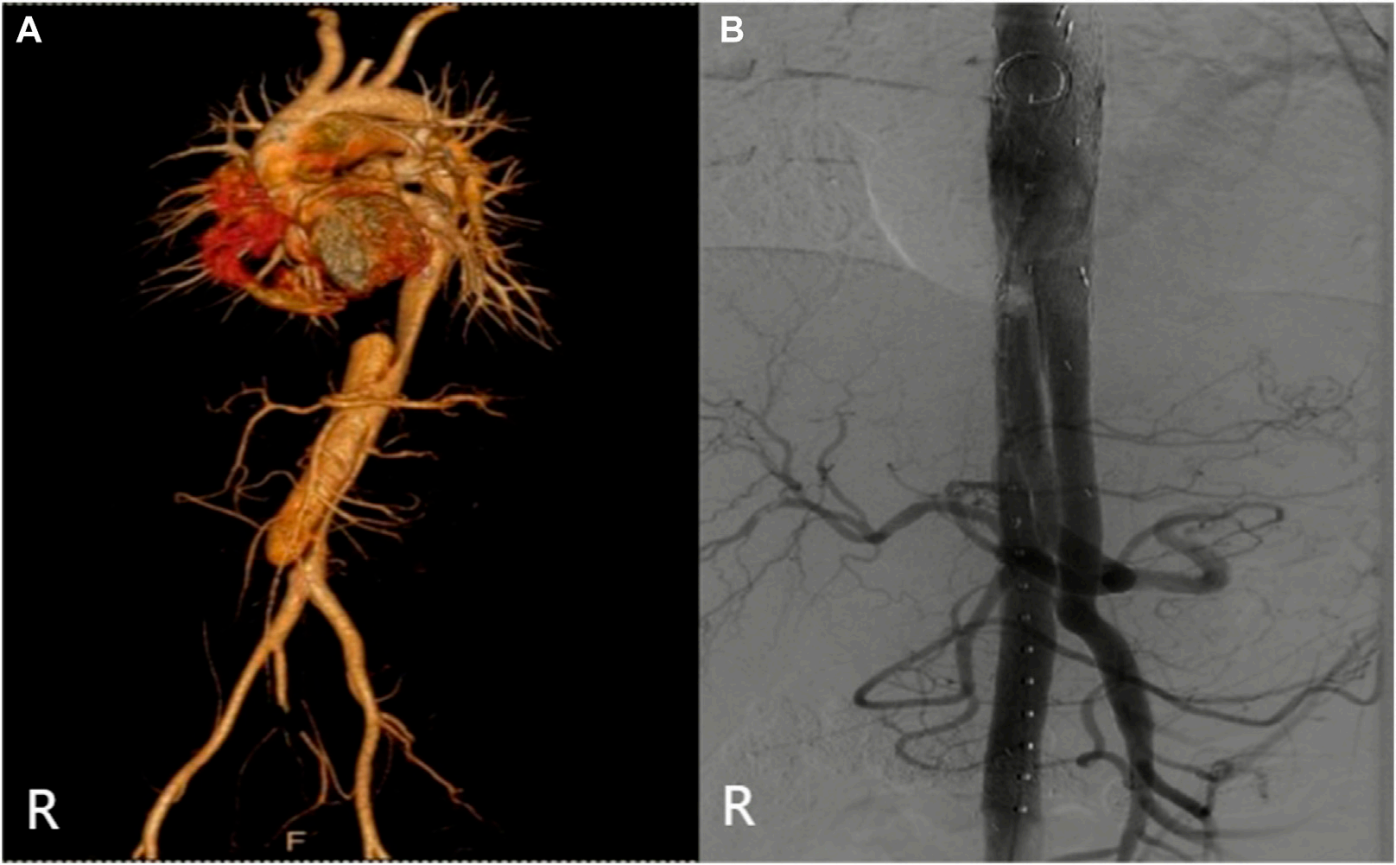
(Case 6) **(A)** Preoperat CTA. **(B)** Intraoperative imaging.

The specific clinical data of the patients are shown in Table 1: six patients (three males) underwent the optimized Octopus operation. The average age was 55.1 ± 17.2 years. CTA was performed in all cases before the operation (Figures 2–7); two cases were diagnosed as pararenal aortic aneurysms, and four were aortic dissections involving the visceral artery. Two cases had a history of endovascular repair of aortic dissection.

The operation plan is shown in Table 2: cases 1–5 all chose the optimized Octopus scheme, that is, embolized CA and reconstructed the bilateral renal artery (BRA) + SMA to thoroughly evaluate the probability of intestinal ischemia and ensure the safety of patients. In case 1, embolization failed because of the stenosis of the CA orifice. Case 6 was a uremic patient with regular dialysis for many years, so the surgical scheme was to reconstruct only CA and SMA. Seventeen visceral arteries were reconstructed, and five CA and two internal iliac arteries were embolized.

**TABLE 2:** Surgical scheme.

Case	Preoperative plan	Postoperative plan
1	Embolization of CA and LIIA, reconstruction of BRA + SMA	Failure of CA embolization, embolization of LIIA, reconstruction of BRA + SMA
2	Embolization of CA, reconstruction of BRA + SMA	Embolization of CA, reconstruction of BRA + SMA
3	Embolization of CA and RIIA, reconstruction BRA + SMA	Embolization of CA and RIIA, reconstruction of BRA + SMA
4	Embolization of CA, reconstruction of BRA + SMA	Embolization of CA, reconstruction of BRA + SMA
5	Embolization of CA, reconstruction of BRA + SMA	Embolization of CA, reconstruction of BRA + SMA
6	Reconstruction of CA + SMA	Reconstruction of CA + SMA

CA, celiac artery; BRA, bilateral renal artery; SMA, superior mesenteric artery; RIIA, right internal iliac artery; LIIA, left internal iliac artery.

The relevant information during the operation is shown in Table 3: the median blood loss was 400 mL (115–975 mL), and the median blood transfusion was 1,215 mL (800–1602.5 mL). Two cases died during the perioperative period, three cases of gutter endoleak were found during the operation (Figures 3, 5, 6), and the renal function of the two cases increased slightly after the operation (basically returned to normal at discharge). Other complications, such as intestinal ischemia and spinal cord ischemia, did not occur. Case 5, whose general condition was poor after the operation, died of respiratory and cardiac arrest 1 day after being transferred to ICU. Case 6, in poor condition after the operation, died of heart failure 2 days after being transferred to the ICU.

**TABLE 3:** Information related to surgery.

Case	Operation time–h	Bleeding volume–ml	Blood transfusion volume–ml	Internal leakage	Other complications	Death
1	4.3	120	1610	No	—	No
2	5	1,1500	1530	Gutter endoleak	Slight increase in creatinine after the operation	No
3	4.3	800	900	No	Slight increase in creatinine after the operation	No
4	5.9	100	800	Gutter endoleak	—	No
5	7.3	400	800	Gutter endoleak	—	Yes
6	5	400	1600	No	—	Yes

Follow-up ranged from 6 months to 30 months

### Follow-up information

The median follow-up period was 14 months. Case 4 died of gastrointestinal bleeding 6 months later. No new endoleaks were found during the follow-up period. In case 2, CTA showed that the endoleak disappeared in the sixth month. The patency rate of the visceral artery was 100%, and no spinal cord ischemia, intestinal ischemia, and so on occurred during the follow-up period. Follow-up information is shown in Table 4; Figures 2–7.

**TABLE 4:** Follow-up information.

Case	Intraoperative internal leakage	Follow-up time (CTA or US)				
		The 1st week	The 6th month	The 12th month	The 24th month	Last follow-up (telephone)
1	No	Good	Good		Good	Good (30 months)
2	Gutter endoleak		Internal leakage disappears			Good (12 months)
3	No			Good (US)		Good (16 months)
4	Gutter endoleak	Internal leakage reduction	Death from gastrointestinal bleeding			

CTA, computed tomographic angiography; US, ultrasound.

## Discussion

Endovascular treatment of aortic lesions involving visceral arteries remains tough. Similar stent technology can be used in patients who cannot use fenestrated and branched stent grafts or are unsuitable for open surgery, including chimneys, periscopes, sandwiches, etc. In Kasirajan (2011) first put forward the concept of the Octopus. Two parallel Gore Excluder bifurcation stents are placed in the descending aorta to complete the stent implantation of the visceral artery. In Silveira et al. (2012) reported the classic Octopus surgery, in which a Gore Excluder bifurcation stent was placed in the descending aorta, and three Viabahn stents were implanted into the short leg of the bifurcation stent to reconstruct CA, SMA, and RRA. The LRA was reclaimed through the long leg of the bifurcation stent. The classic Octopus operation is challenging; the operation time is very long, more blood is lost, and the corresponding risk is much higher. Therefore, at present, it is more usual to use the optimized Octopus technology, that is, based on ensuring the safety of patients, embolization of CA, and short leg reconstruction of SMA and RRA. When the diameter of the SMA is more significant than 8 mm, the SMA can be reconstructed in the short leg, and the BRA can be reconstructed by the extended leg and periscope techniques. Compared with the classic Octopus technique, the optimized Octopus scheme can provide more stent combination schemes to meet the visceral artery’s anatomical requirements while reducing the gutter endoleak as much as possible. Indeed, in the current research, the optimized Octopus scheme is better than the classic Octopus scheme in terms of operation time and blood loss (Wang et al., 2023).

Octopus surgery requires the concerted efforts of a team of experienced vascular surgeons, so it is by no means a simple one-person show. Before an operation, the CTA data of patients must be measured and evaluated in detail, and the operation plan should be worked out, verified, and discussed repeatedly. To ensure the safety and feasibility of the operation, we chose the optimized Octopus scheme to reduce the operation’s difficulty, the operation time, and the error rate. The average operation time of our operation is 5.3 h. The average operation time of 10 cases of TAAA and pararenal aortic aneurysms treated by Wang et al. (2023) with the Octopus technique was 7.8 h. The average operation time of 21 cases of TAAA and aortic dissection treated by Dua et al. (2019) with the Octopus technique was 8 hours. The average operation time of 11 cases of TAAA and aortic dissection treated by Hsu et al. (2019) with the Octopus technique was 9.4 h. Compared with other people’s data, our average operating time is significantly lower than their operating times. Long-time operations are a test for doctors’ physical strength and energy, and the whole operation requires a high degree of concentration, especially at the moment of stent release because positioning is crucial. This requires experienced and tacit cooperation with the team; if there is a mistake in the positioning process of stent implantation, it will cause severe or irreparable consequences. Therefore, although the patient’s essential physical condition is a prerequisite for completing the operation, an experienced vascular surgery team is another critical factor in completing the operation.

When using the Octopus technique to reconstruct visceral branches, it is essential to ensure that the reconstructed visceral artery blood flow meets the physiological requirements. Xu et al. (2019) studied the hemodynamics of specific patients before and after Octopus surgery. The results showed that extending the 4 cm at the head end of the Gore Excluder bifurcation stent and reducing the 2 mm bifurcation stent legs were more in line with the physiological requirements. This study only analyzed specific patients, and there are few such studies, so many related studies are needed to confirm it in the future.

Gutter endoleak is a prevalent complication in Octopus surgery. Franklin et al. (2016) studied the diameter of three Viabahn stents embedded in Gore Excluder bifurcation stent short leg (*D* = 13 mm). The results showed that when the short leg was implanted with Viabahn stents with a diameter of 8 mm or 7 mm (specific scheme: 8 mm + 8 mm + 8 mm or 8 mm + 8 mm + 7 mm or 8 mm + 7 mm + 7 mm), the stent gap was the smallest and the incidence of endoleak was the lowest, and balloon dilatation was not recommended in this study, which would damage the compliance of stents, increase the gap between stents, and increase the incidence of endoleak. Because our surgical scheme chose the optimized Octopus scheme with two Viabahn stents in the short leg, it is unsuitable for the above research scheme. We chose the stent diameter scheme: π*D* + 2*D* = π*D*_1_ + π*D*_2_. *D* is the distal diameter of the iliac leg-covered stent containing two Viabahn stents, and *D*_1_ and *D*_2_ are the diameters of the SMA and RRA, respectively. The diameters of the SMA and RRA were measured using CTA before the operation. The value of *D* was calculated according to the formula, from which the distal diameter of the iliac leg was selected to reduce the occurrence of endoleak as much as possible. Although gutter endoleak has been well studied, it cannot be completely avoided, which may lead to the growth of aneurysms, especially in the case of ruptured aneurysms. However, gutter endoleak also has its bright side. One of the severe complications of the Octopus operation is spinal cord ischemia and even paraplegia, which is a devastating disaster for patients. Because gutter endoleak has a specific blood supply to the spinal cord, it can reduce the incidence of postoperative paraplegia. The mild gutter endoleak found after intraoperative angiography does not need to be embolized immediately. The self-healing rate of mild gutter endoleak is very high, so it can be observed and gain time for establishing collateral circulation of the spinal cord. After the operation, our patients’ CTA examination showed that all gutter endoleaks disappeared spontaneously, and no secondary intervention was needed.

Hsu et al. (2019) reported using the Octopus technique to treat 11 cases of TAAA and aortic dissection from June 2014 to June 2017. The incidence of major complications was 45.5%, including three deaths, one permanent paraplegia, and one temporary paraplegia. Wooster et al. (2018) reported using the Octopus technology to treat eight cases of TAAA from January 2015 to January 2017. The incidence of major complications was 37.5%; two cases died during the perioperative period, and one case of temporary paraplegia. Dua et al. (2019) reported that the Octopus technology was used to treat 21 cases of TAAA and aortic dissection from 2015 to 2018. Perioperative complications included 24% acute renal injury, 19% paraplegia, 19% prolonged mechanical ventilation, 14.2% perioperative mortality, 4.9% myocardial infarction, and 4.8% intestinal ischemia. The 1-month survival rate was 90.5%, the 6-month survival rate was 88.3%, the 1-year survival rate was 71.4%, and the 3-year survival rate was 52.1%. Wang et al. (2023) reported 10 cases of TAAA and pararenal aortic aneurysms treated with the Octopus technique from May 2016 to May 2019. There were no perioperative deaths. One patient died of lung cancer at 14 months. During the follow-up period, the patency rate of branches was 90% (28/31). All three occlusive vessels were renal arteries. Other people, such as Xiong et al. (2017), Li et al. (2018), have reported cases of Octopus, and the results of the operation and prognosis are also very successful. Based on the above, we seem to conclude that Octopus technology is becoming more and more mature, and the prognosis of patients is improving. However, this may not be the case. The results of the six optimized Octopus operations we performed from August 2020 to February 2022 were not as good as we expected. Although the technical success rate was 100% (17/17), the clinical success rate was only 66.7% (4/6). Half a year later, one patient died of gastrointestinal bleeding, and the half-year survival rate was only 50% (3/6). We are cautious about such a result.

Our patients were followed up from 6 to 27 months (Table 4). Intraoperative internal leakage was 50% (3/6), and three cases were mild gutter endoleak. Postoperative CTA internal leakage disappeared spontaneously. There were no other complications during the follow-up period, such as stent occlusion, displacement, spinal cord ischemia, or intestinal ischemia. Our other complications were well treated, but the half-year survival rate is only 50%. The survival rate is the most crucial parameter in judging the mode of operation, so we need to be cautious about this promising Octopus technology. As is well known, in the case of the vast majority of people publishing papers without reporting bad news, many failed cases have yet to be reported. Therefore, Octopus technology needs continuous optimization, improvement, and longer follow-up.

## Conclusion

With fenestrated and branched stent grafts technology not widely available, and off label use not a viable option, Octopus technology for treating complex aortic lesions should be considered. There is no doubt that Octopus technology is an up-and-coming surgical method, but we should recognize its difficulty in operation-related complications, and long-term prognosis. We should pay attention to and continue to optimize Octopus regimens. In the future, we can only popularize this technology and benefit more patients by further reducing the difficulty of the Octopus technology, the incidence of complications, and improving the long-term survival rate.

## Data Availability

The original contributions presented in the study are included in the article/supplementary material, further inquiries can be directed to the corresponding author.
